# A new result on the existence of periodic solutions for Liénard equations with a singularity of repulsive type

**DOI:** 10.1186/s13660-016-1285-8

**Published:** 2017-02-07

**Authors:** Shiping Lu

**Affiliations:** College of Math & Statistics, Nanjing University of Information Science and Technology, Nanjing, 210044 China

**Keywords:** 34C25, 34B16, 34B18, Liénard equation, topological degree, singularity, periodic solution

## Abstract

In this paper, the problem of the existence of a periodic solution is studied for the second order differential equation with a singularity of repulsive type

$$x''(t)+f\bigl(x(t)\bigr)x'(t)-g\bigl(x(t) \bigr)+\varphi(t)x(t)=h(t), $$

where $g(x)$ is singular at $x=0$, *φ* and *h* are *T*-periodic functions. By using the continuation theorem of Manásevich and Mawhin, a new result on the existence of positive periodic solution is obtained. It is interesting that the sign of the function $\varphi(t)$ is allowed to change for $t\in[0,T]$.

## Introduction

The aim of this paper is to search for positive *T*-periodic solutions for a second order differential equation with a singularity in the following form:

1.1 $$ x''(t)+f\bigl(x(t)\bigr)x'(t)-g \bigl(x(t)\bigr)+\varphi(t)x(t)=h(t), $$

where $f:[0,\infty)\rightarrow R$ is an arbitrary continuous function, $g\in C((0,+\infty), (0,+\infty))$, and $g(x)$ is singular of repulsive type at $x=0$, *i.e.*, $g(x)\rightarrow+\infty$ as $x\rightarrow 0^{+}$, $\varphi,h: R\rightarrow R$ are *T*-periodic functions with $h\in L^{2}([0,T], R)$ and $\varphi\in C([0,T], R)$, and the sign of the function *φ* is allowed to change for $t\in[0,T]$.

The study of the problem of periodic solutions to scalar equations with a singularity began with work of Forbat and Huaux [1, 2], where the singular term in the equations models the restoring force caused by a compressed perfect gas (see [3–6] and the references therein). In the past years, many works used the methods, such as the approaches of critical point theory [7–12], the techniques of some fixed point theorems [13–15], and the approaches of topological degree theory, in particular, of some continuation theorems of Mawhin (see [6, 16–22]), to study the existence of positive periodic solutions for some second order ordinary differential equations with singularities. For example, in [15], by using a fixed point theorem in cones, the existence of positive periodic solutions to equation (1.1) was investigated for the conservative case, *i.e.*, $f(x)\equiv0$. But the function $\varphi(t)$ is required to be $\varphi(t)\ge0$ for all $t\in [0,T]$. The method of topological degree theory, together with the technique of upper and lower solutions, was first used by Lazer and Solimini in the pioneering paper [18] for considering the problem of a periodic solution to a second order differential equations with singularities. Jebelean and Mawhin in [6] considered the problem of a *p*-Laplacian Liénard equation of the form

1.2 $$ \bigl(\bigl\vert x'\bigr\vert ^{p-2}x' \bigr)'+f(x)x'+g(x)=h(t) $$

and

1.3 $$ \bigl(\bigl\vert x'\bigr\vert ^{p-2}x' \bigr)'+f(x)x'-g(x)=h(t), $$

where $p>1$ is a constant, $f: [0,+\infty)\rightarrow R$ is an arbitrary continuous function, $h: R\rightarrow R$ is a *T*-periodic function with $h\in L^{\infty}([0,T],R)$, $g: (0,+\infty)\rightarrow (0,+\infty)$ is continuous, $g(x)\rightarrow+\infty$ as $x\rightarrow 0^{+}$. They extended the results of Lazer and Solimini in [16] to equation (1.2) and equation (1.3). For equation (1.3), the crucial condition is that the function $g(x)$ is bounded, which means that equation (1.3) is not singular at $x=+\infty$.

By using a continuation theorem of Mawhin, Zhang in [18] studied the problem of periodic solutions of the Liénard equation with a singularity of repulsive type,

1.4 $$ x''+f(x)x'+g(t,x)=0, $$

where $f: {R}\rightarrow{R}$ is continuous, $g: {R}\times(0,+\infty )\rightarrow{R}$ is an $L^{2}$-Carathéodory function with *T*-periodic in the first argument, and it is singular at $x=0$, *i.e.*, $g(t,x)$ is unbounded as $x\rightarrow0^{+}$. Different from the equation studied in [6, 16], which is only singular at $x=0$, equation (1.4) is provided with both singularities at $x=+\infty$ and at $x=0$. In [19], Wang further studied the existence of positive periodic solutions for a delay Liénard equation with a singularity of repulsive type

1.5 $$ x''+f(x)x'+g\bigl(t,x(t- \tau)\bigr)=0. $$

In [18, 19], the following balance condition between the singular force at the origin and at infinity is needed.

(h_1_) There exist constants $0< D_{1}< D_{2}$ such that if *x* is a positive continuous *T*-periodic function satisfying

$$\int_{0}^{T} g\bigl(t,x(t)\bigr)\,dt=0, $$

then

1.6 $$ D_{1}\leq x(\tau)\leq D_{2},\quad \mbox{for some } \tau\in[0,T]. $$

From the proof of [18, 19], we see that the balance condition (h_1_) is crucial for estimating *a priori bounds* of periodic solutions. Now, the question is how to investigate the existence of positive periodic solutions for the equations like equation (1.4) or equation (1.5) without the balance condition (h_1_).

Motivated by this, in this paper, we study the existence of positive *T*-periodic solutions for equation (1.1) under the condition that the sign of the function *φ* is allowed to change for $t\in [0,T]$. For this case, the balance condition (h_1_) may not be satisfied. By using the continuation theorem of Manásevich and Mawhin, a new result on the existence of positive periodic solutions is obtained.

## Preliminary lemmas

Throughout this paper, let $C_{T}=\{x\in C(R,R): x(t+T)= x(t) \mbox{ for all } t\in R\}$ with the norm defined by $|x|_{\infty}=\max_{t\in [0,T]}|x(t)|$. For any *T*-periodic solution $y(t)$ with $y\in L^{1}([0,T], R)$, $y_{+}(t)$ and $y_{-}(t)$ denote $\max\{y(t),0\}$ and $-\min\{y(t),0\}$, respectively, and $\bar{y}=\frac{1}{T}\int _{0}^{T}y(s)\,ds$. Clearly, $y(t)=y_{+}(t)-y_{-}(t)$ for all $t\in R$, and $\bar {y}=\overline{y_{+}}-\overline{y_{-}}$.

The following lemma is a consequence of Theorem 3.1 in [23].

### Lemma 1

*Assume that there exist positive constants* $M_{0}$, $M_{1}$, *and* $M_{2}$ *with* $0< M_{0}< M_{1}$, *such that the following conditions hold*.

1. *For each* $\lambda\in(0,1]$, *each possible positive* *T*-*periodic solution* *x* *to the equation*
  $$u''+\lambda f(u)u'-\lambda g(u)+\lambda \varphi(t)u=\lambda h(t) $$
  *satisfies the inequalities* $M_{0}< x(t)< M_{1}$ *and* $|x'(t)|< M_{2}$ *for all* $t\in[0,T]$.
2. *Each possible solution* *c* *to the equation*
  $$g(c)-c\bar{\varphi}+\bar{h}=0 $$
  *satisfies the inequality* $M_{0}< c< M_{1}$.
3. *We have*
  $$\bigl(g(M_{0})-\bar{\varphi}M_{0}+\bar{h} \bigr) \bigl(g(M_{1})-\bar{\varphi }M_{1}+\bar{h} \bigr)< 0. $$
  *Then equation* (1.1) *has at least one* *T*-*periodic solution* *u* *such that* $M_{0}< u(t)< M_{1}$ *for all* $t\in[0,T]$.

### Lemma 2

[19]

*Let* *x* *be a continuous* *T*-*periodic continuously differential function*. *Then*, *for any* $\tau\in(0,T]$,

$$\biggl( \int_{0}^{T}\bigl\vert x(s)\bigr\vert ^{2}\,ds \biggr)^{1/2}\le \frac{T}{\pi} \biggl( \int_{0}^{T}\bigl\vert x'(s)\bigr\vert ^{2}\,ds \biggr)^{1/2}+ \sqrt{T}\bigl\vert x(\tau)\bigr\vert . $$

In order to study the existence of positive periodic solutions to equation (1.1), we list the following assumptions.

[H_1_] The function $\varphi(t)$ satisfies the following conditions:

$$\int_{0}^{T}\varphi_{+}(s)\,ds>0, \qquad \sigma:= \frac{\int_{0}^{T}\varphi_{-}(s)\,ds}{\int _{0}^{T}\varphi_{+}(s)\,ds}\in[0,1) $$

and

$$\sigma_{1}:=\frac{T}{\pi}|\varphi_{+}|_{\infty}^{1/2}+ \frac{T^{\frac {1}{2}} (\int_{0}^{T}\varphi_{-}(s)^{2}\,ds )^{\frac{1}{2}}}{\int _{0}^{T}\varphi_{+}(s)\,ds}\in(0,1); $$

[H_2_] there are constants $A>0$ and $M>0$ such that $g(x)\in(0,A)$ for all $x>M$;

[H_3_] $\int_{0}^{1}g(s)\,ds=+\infty$.

### Remark 1

If assumptions [H_1_]-[H_2_] hold, then there are constants $D_{1}$ and $D_{2}$ with $0< D_{1}< D_{2}$ such that

$$g(x)-\bar{\varphi}x+\bar{h}>0 \quad\mbox{for all } x\in(0,D_{1}) $$

and

$$g(x)-\bar{\varphi}x+\bar{h}< 0 \quad\mbox{for all } x\in(D_{2},\infty). $$

Furthermore, assumption $\sigma_{1}\in(0,1)$ in [H_1_] is different from the corresponding condition $\int_{0}^{T}\varphi_{+}(s)\,ds<\frac{4}{T}$ in [20].

Now, we suppose that assumptions [H_1_] and [H_2_] hold, and we embed equation (1.1) into the following equation family with a parameter $\lambda\in(0,1]$:

2.1 $$ x''+\lambda f(x)x'-\lambda g(x)+\lambda\varphi(t)x=\lambda h(t),\quad \lambda\in(0,1]. $$

Let

$$\varOmega =\bigl\{ x\in C_{T}: x''+\lambda f(x)x'-\lambda g(x)+\lambda\varphi (t)x=\lambda h(t), \lambda \in(0,1]; x(t)>0, \forall t\in[0,T]\bigr\} , $$

and

2.2 $$ M_{0}=\frac{ (\int_{0}^{T}\varphi _{-}(s)^{2}\,ds )^{\frac{1}{2}}}{\int_{0}^{T}\varphi_{+}(s)\,ds}A_{0}^{2}+ \frac {A+|\bar{h}|}{\overline{\varphi_{+}}}+|\varphi_{+}|_{\infty}A_{0}^{2} T^{\frac {1}{2}}+A_{0}T^{\frac{1}{2}} \biggl( \int_{0}\bigl|h_{-}(t)\bigr|^{2}\,dt \biggr)^{\frac{1}{4}}, $$

where

$$A_{0}=\frac{T}{\pi(1-\sigma_{1})} \biggl( \int_{0}^{T}\bigl|h_{-}(t)\bigr|^{2}\,dt \biggr)^{\frac {1}{4}}+ \biggl(\frac{(A+|\bar{h}|)T^{\frac{1}{2}}}{(1-\sigma_{1})\overline {\varphi_{+}}} \biggr)^{\frac{1}{2}}, $$

$A>0$ is a constant determined by assumption [H_2_]. Clearly, $M_{0}$ and $A_{0}$ are all independent of $(\lambda,x)\in(0,1]\times \varOmega $. Let $M>0$ be determined by assumption [H_2_], then there is a positive integer $k_{0}$ such that

2.3 $$ k_{0}M\ge M_{0}. $$

### Lemma 3

*Assume that assumptions* [H_1_]-[H_2_] *hold*, *then there is an integer* $k^{*}>k_{0}$ *such that*, *for each function* $u\in \varOmega $, *there is a point* $t_{0}\in[0,T]$ *satisfying*

$$u(t_{0})\le k^{*}M. $$

### Proof

If the conclusion does not hold, then, for each $k>k_{0}$, there is a function $u_{k}\in \varOmega $ satisfying

2.4 $$ u_{k}(t)>kM \quad \mbox{for all } t\in[0,T]. $$

From the definition of *Ω*, we see

2.5 $$ u_{k}''+\lambda f(u_{k})u_{k}'-\lambda g(u_{k})+ \lambda\varphi(t)u_{k}=\lambda h(t), \quad\lambda\in(0,1], $$

and by using assumption [H_2_],

2.6 $$ 0< g\bigl(u_{k}(t)\bigr)< A, \quad\mbox{for all } t \in[0,T]. $$

By integrating equation (2.5) over the interval $[0,T]$, we have

$$\int_{0}^{T}\varphi(t)u_{k}(t)\,dt= \int_{0}^{T}g\bigl(u_{k}(t)\bigr)\,dt+ \int_{0}^{T}h(t)\,dt, $$

*i.e.*,

$$\int_{0}^{T}\varphi_{+}(t)u_{k}(t)\,dt= \int_{0}^{T}\varphi_{-}(t)u_{k}(t)\,dt+ \int _{0}^{T}g\bigl(u_{k}(t)\bigr)\,dt+ \int_{0}^{T}h(t)\,dt. $$

Since $\varphi_{+}(t)\ge0$ and $\varphi_{-}(t)\ge0$ for all $t\in[0,T]$, it follows from the integral mean value theorem that there is a point $\xi\in[0,T]$ such that

$$\begin{aligned} u_{k}(\xi) \int_{0}^{T}\varphi_{+}(t)\,dt =& \int_{0}^{T}\varphi _{-}(s)u_{k}(s)\,ds+ \int_{0}^{T}g\bigl(u_{k}(t)\bigr)\,dt+T \bar{h} \\ \le& \biggl( \int_{0}^{T}\varphi_{-}(s)^{2}\,ds \biggr)^{\frac{1}{2}} \biggl( \int _{0}^{T}\bigl|u_{k}(s)\bigr|^{2}\,ds \biggr)^{\frac{1}{2}}+ \int_{0}^{T}g\bigl(u_{k}(t)\bigr)\,dt+T \bar{h}, \end{aligned}$$

which together with (2.6) yields

2.7 $$ u_{k}(\xi)< \frac{ (\int_{0}^{T}\varphi _{-}(s)^{2}\,ds )^{\frac{1}{2}}}{\int_{0}^{T}\varphi_{+}(s)\,ds} \biggl( \int _{0}^{T}u_{k}(s)^{2}\,ds \biggr)^{\frac{1}{2}}+\frac{A+|\bar{h}|}{\overline{\varphi_{+}}}. $$

It follows from $|u_{k}|_{\infty}\le u_{k}(\xi)+T^{\frac{1}{2}} (\int _{0}^{T}|u_{k}'(s)|^{2}\,ds )^{\frac{1}{2}}$ that

2.8 $$ |u_{k}|_{\infty}\le\frac{ (\int _{0}^{T}\varphi_{-}(s)^{2}\,ds )^{\frac{1}{2}}}{\int_{0}^{T}\varphi_{+}(s)\,ds} \biggl( \int_{0}^{T}u_{k}(s)^{2}\,ds \biggr)^{\frac{1}{2}}+\frac{A+\bar{h}}{\overline {\varphi_{+}}}+T^{\frac{1}{2}} \biggl( \int_{0}^{T}\bigl\vert u_{k}'(s) \bigr\vert ^{2}\,ds \biggr)^{1/2}. $$

On the other hand, by multiplying equation (2.5) with $u_{k}(t)$, and integrating it over the interval $[0,T]$, we obtain

$$\int_{0}^{T}\bigl\vert u_{k}'(t) \bigr\vert ^{2}\,dt=-\lambda \int_{0}^{T}g\bigl(u_{k}(t) \bigr)u_{k}(t)\,dt+\lambda \int _{0}^{T}\varphi(t)u_{k}^{2}(t)\,dt- \lambda \int_{0}^{T}h(t)u_{k}(t)\,dt, $$

which together with the fact of $g(x)>0$ for all $x>0$ gives

$$\begin{aligned} \int_{0}^{T}\bigl\vert u_{k}'(t) \bigr\vert ^{2}\,dt < & \lambda \int_{0}^{T}\varphi _{+}(t)u_{k}^{2}(t)\,dt+ \lambda \int_{0}^{T}h_{-}(t)u_{k}(t)\,dt \\ \le&|\varphi_{+}|_{\infty} \int_{0}^{T}\bigl\vert u_{k}(t)\bigr\vert ^{2}\,dt+ \biggl( \int _{0}^{T}\bigl\vert u_{k}(t)\bigr\vert ^{2}\,dt \biggr)^{\frac{1}{2}} \biggl( \int_{0}^{T}\bigl|h_{-}(t)\bigr|^{2}\,dt \biggr)^{\frac{1}{2}}, \end{aligned}$$

*i.e.*,

2.9 $$\begin{aligned} & \biggl( \int_{0}^{T}\bigl|u_{k}'(t)\bigr|^{2}\,dt \biggr)^{1/2} \\ &\quad< |\varphi_{+}|_{\infty}^{1/2} \biggl( \int_{0}^{T}\bigl|u_{k}(t)\bigr|^{2}\,dt \biggr)^{\frac {1}{2}}+ \biggl( \int_{0}^{T}\bigl|u_{k}(t)\bigr|^{2}\,dt \biggr)^{\frac{1}{4}} \biggl( \int _{0}^{T}\bigl|h_{-}(t)\bigr|^{2}\,dt \biggr)^{\frac{1}{4}}. \end{aligned}$$

By using Lemma 2, we have

$$\biggl( \int_{0}^{T}\bigl|u_{k}(s)\bigr|^{2}\,ds \biggr)^{1/2}\le \frac{T}{\pi} \biggl( \int_{0}^{T}\bigl|u_{k}'(s)\bigr|^{2}\,ds \biggr)^{1/2}+\sqrt{T}\bigl|u_{k}(\xi)\bigr|. $$

Substituting (2.7) and (2.9) into the above formula,

$$\begin{aligned} & \biggl( \int_{0}^{T}\bigl|u_{k}(t)\bigr|^{2}\,dt \biggr)^{1/2} \\ &\quad< \frac {T}{\pi} \biggl[|\varphi_{+}|_{\infty}^{1/2} \biggl( \int_{0}^{T}\bigl|u_{k}(t)\bigr|^{2}\,dt \biggr)^{\frac{1}{2}}+ \biggl( \int_{0}^{T}\bigl|u_{k}(t)\bigr|^{2}\,dt \biggr)^{\frac{1}{4}} \biggl( \int_{0}^{T}\bigl|h_{-}(t)\bigr|^{2}\,dt \biggr)^{\frac{1}{4}} \biggr] \\ &\qquad{}+ \frac{T^{\frac{1}{2}} (\int_{0}^{T}\varphi_{-}(s)^{2}\,ds )^{\frac {1}{2}}}{\int_{0}^{T}\varphi_{+}(s)\,ds} \biggl( \int_{0}^{T}u_{k}(s)^{2}\,ds \biggr)^{\frac {1}{2}}+\frac{(A+\bar{h})T^{\frac{1}{2}}}{\overline{\varphi_{+}}} \\ &\quad=\sigma_{1} \biggl( \int_{0}^{T}\bigl|u_{k}(t)\bigr|^{2}\,dt \biggr)^{\frac{1}{2}}+\frac{T}{\pi } \biggl( \int_{0}^{T}\bigl|h_{-}(t)\bigr|^{2}\,dt \biggr)^{\frac{1}{4}} \biggl( \int _{0}^{T}\bigl|u_{k}(t)\bigr|^{2}\,dt \biggr)^{\frac{1}{4}}+\frac{(A+|\bar{h}|)T^{\frac {1}{2}}}{\overline{\varphi_{+}}}, \end{aligned}$$

where

$$\sigma_{1}=\frac{T}{\pi}|\varphi_{+}|_{\infty}^{1/2}+ \frac{T^{\frac {1}{2}} (\int_{0}^{T}\varphi_{-}(s)^{2}\,ds )^{\frac{1}{2}}}{\int _{0}^{T}\varphi_{+}(s)\,ds}\in(0,1), $$

which is determined by assumption [H_1_]. This gives

2.10 $$\begin{aligned} & \biggl( \int_{0}^{T}\bigl|u_{k}(t)\bigr|^{2}\,dt \biggr)^{1/2} \\ &\quad\le \frac{T}{\pi(1-\sigma_{1})} \biggl( \int_{0}^{T}\bigl|h_{-}(t)\bigr|^{2}\,dt \biggr)^{\frac {1}{4}} \biggl( \int_{0}^{T}\bigl|u_{k}(t)\bigr|^{2}\,dt \biggr)^{\frac{1}{4}}+\frac{(A+|\bar {h}|)T^{\frac{1}{2}}}{(1-\sigma_{1})\overline{\varphi_{+}}}, \end{aligned}$$

*i.e.*,

2.11 $$ \biggl( \int_{0}^{T}\bigl\vert u_{k}(t)\bigr\vert ^{2}\,dt \biggr)^{\frac {1}{4}}\le A_{0}, $$

where

$$A_{0}=\frac{T}{\pi(1-\sigma_{1})} \biggl( \int_{0}^{T}\bigl|h_{-}(t)\bigr|^{2}\,dt \biggr)^{\frac {1}{4}}+ \biggl(\frac{(A+|\bar{h}|)T^{\frac{1}{2}}}{(1-\sigma_{1})\overline {\varphi_{+}}} \biggr)^{\frac{1}{2}}. $$

It follows from (2.9) that

2.12 $$ \biggl( \int_{0}^{T}\bigl|u_{k}'(t)\bigr|^{2}\,dt \biggr)^{1/2}< |\varphi_{+}|_{\infty}A_{0}^{2}+A_{0} \biggl( \int_{0}^{T}\bigl\vert h_{-}(t)\bigr\vert ^{2}\,dt \biggr)^{\frac{1}{4}}. $$

Substituting (2.11)-(2.12) into (2.8), we have

$$|u_{k}|_{\infty}< \frac{ (\int_{0}^{T}\varphi_{-}(s)^{2}\,ds )^{\frac {1}{2}}}{\int_{0}^{T}\varphi_{+}(s)\,ds}A_{0}^{2}+ \frac{A+|\bar{h}|}{\overline {\varphi_{+}}}+|\varphi_{+}|_{\infty}A_{0}^{2} T^{\frac{1}{2}}+A_{0}T^{\frac {1}{2}} \biggl( \int_{0}^{T}\bigl|h_{-}(t)\bigr|^{2}\,dt \biggr)^{\frac{1}{4}}, $$

which together with (2.2) yields

2.13 $$ u_{k}(t)< M_{0} \quad\mbox{for all } t \in[0,T]. $$

By the definition of $k_{0}$, we see from (2.3) that (2.13) contradicts (2.4). This contradiction implies that the conclusion of Lemma 3 is true. □

## Main results

### Theorem 1

*Assume that* [H_1_]-[H_3_] *hold*. *Then equation* (1.1) *has at leat one positive* *T*-*periodic solution*.

### Proof

Firstly, we will show that there exist $M_{1},M_{2}$ with $M_{1}>k^{*}M$ and $M_{2}>0$ such that each positive *T*-periodic solution $u(t)$ of equation (2.1) satisfies the inequalities

3.1 $$ u(t)< M_{1},\qquad \bigl|u'(t)\bigr|< M_{2}, \quad\mbox{for all } t\in[0,T]. $$

In fact, if *u* is an arbitrary positive *T*-periodic solution of equation (2.1), then

3.2 $$ u''+\lambda f(u)u'-\lambda g(u)+\lambda\varphi(t)u=\lambda h(t),\quad \lambda\in(0,1]. $$

This implies $u\in \varOmega $. So by using Lemma 3 that there is a point $t_{0}\in[0,T]$ such that

3.3 $$ u(t_{0})\le k^{*}M, $$

and then

3.4 $$ |u|_{\infty}\le k^{*}M+T^{1/2} \biggl( \int _{0}^{T}\bigl|u'(s)\bigr|^{2}\,ds \biggr)^{1/2}. $$

Integrating (3.2) over the interval $[0,T]$, we have

3.5 $$ - \int_{0}^{T} g\bigl(u(t)\bigr)\,dt+ \int_{0}^{T}\varphi (t)u(t)\,dt= \int_{0}^{T}h(t)\,dt. $$

Since $g(x)\rightarrow+\infty$ as $x\rightarrow0^{+}$, we see from (3.5) that there is a point $t_{1}\in[0,T]$ such that

3.6 $$ u(t_{1})\ge\gamma, $$

where $\gamma< k^{*}M$ is a positive constant, which is independent of $\lambda\in(0,1]$. Similar to the proof of (2.9), we have

3.7 $$\begin{aligned} & \biggl( \int_{0}^{T}\bigl|u'(t)\bigr|^{2}\,dt \biggr)^{1/2} \\ &\quad< |\varphi _{+}|_{\infty}^{1/2} \biggl( \int_{0}^{T}\bigl|u(t)\bigr|^{2}\,dt \biggr)^{\frac{1}{2}}+ \biggl( \int_{0}^{T}\bigl|u(t)\bigr|^{2}\,dt \biggr)^{\frac{1}{4}} \biggl( \int_{0}^{T}\bigl|h_{-}(t)\bigr|^{2}\,dt \biggr)^{\frac{1}{4}}. \end{aligned}$$

By using Lemma 2, we have

3.8 $$ \biggl( \int_{0}^{T}\bigl|u(s)\bigr|^{2}\,ds \biggr)^{1/2}\le \frac{T}{\pi} \biggl( \int_{0}^{T}\bigl\vert u'(s)\bigr\vert ^{2}\,ds \biggr)^{1/2}+\sqrt{T}\bigl\vert u(t_{0})\bigr\vert , $$

where $t_{0}$ is determined in (3.3). Substituting (3.7) into (3.8), we have

$$\begin{aligned} & \biggl( \int_{0}^{T}\bigl|u(t)\bigr|^{2}\,dt \biggr)^{1/2} \\ &\quad< \frac{T}{\pi} \biggl[|\varphi_{+}|_{\infty}^{1/2} \biggl( \int _{0}^{T}\bigl|u(t)\bigr|^{2}\,dt \biggr)^{\frac{1}{2}}+ \biggl( \int_{0}^{T}\bigl|u(t)\bigr|^{2}\,dt \biggr)^{\frac{1}{4}} \biggl( \int_{0}^{T}\bigl|h_{-}(t)\bigr|^{2}\,dt \biggr)^{\frac{1}{4}} \biggr] \\ &\qquad{} +T^{\frac{1}{2}}k^{*}M \\ &\quad=\frac{T}{\pi}|\varphi_{+}|_{\infty}^{1/2} \biggl( \int_{0}^{T}\bigl|u(t)\bigr|^{2}\,dt \biggr)^{\frac{1}{2}}+\frac{T}{\pi} \biggl( \int_{0}^{T}\bigl|h_{-}(t)\bigr|^{2}\,dt \biggr)^{\frac {1}{4}} \biggl( \int_{0}^{T}\bigl|u(t)\bigr|^{2}\,dt \biggr)^{\frac{1}{4}}+ T^{\frac{1}{2}}k^{*}M, \end{aligned}$$

which results in

3.9 $$\begin{aligned} & \biggl(1- \frac{T}{\pi}|\varphi_{+}|_{\infty }^{1/2} \biggr) \biggl( \int_{0}^{T}\bigl|u(t)\bigr|^{2}\,dt \biggr)^{1/2} \\ &\quad< \frac{T}{\pi} \biggl( \int_{0}^{T}\bigl|h_{-}(t)\bigr|^{2}\,dt \biggr)^{\frac{1}{4}} \biggl( \int_{0}^{T}\bigl|u(t)\bigr|^{2}\,dt \biggr)^{\frac{1}{4}}+ T^{\frac{1}{2}}k^{*}M. \end{aligned}$$

Since $\frac{T}{\pi}|\varphi_{+}|_{\infty}^{1/2}<\sigma_{1}\in(0,1)$, it follows from (3.9) that there is a constant $\rho>0$, which is independent of $\lambda\in(0,1]$, such that

$$\biggl( \int_{0}^{T}\bigl|u(t)\bigr|^{2}\,dt \biggr)^{1/2}< \rho, $$

and then by (3.7), we have

$$\biggl( \int_{0}^{T}\bigl|u'(t)\bigr|^{2}\,dt \biggr)^{1/2}< |\varphi_{+}|_{\infty}^{1/2}\rho + \biggl( \int_{0}^{T}\bigl|h_{-}(t)\bigr|^{2}\,dt \biggr)^{\frac{1}{4}}\rho^{1/2}. $$

It follows from (3.4) that

$$|u|_{\infty}< k^{*}M+T^{1/2}|\varphi_{+}|_{\infty}^{1/2} \rho+(T\rho )^{1/2} \biggl( \int_{0}^{T}\bigl|h_{-}(t)\bigr|^{2}\,dt \biggr)^{\frac{1}{4}}:=M_{1}, $$

*i.e.*,

3.10 $$ u(t)< M_{1}, \quad\mbox{for all } t\in[0,T]. $$

Now, if *u* attains its maximum over $[0,T]$ at $t_{2}\in[0,T]$, then $u'(t_{2})=0$ and we deduce from (3.2) that

$$u'(t)=\lambda \int_{t_{2}}^{t}\bigl[-f(u)u'+g(u)- \varphi(t)u+h(t)\bigr]\,dt $$

for all $t\in[t_{2},t_{2}+T]$. Thus, if $F'=f$, then

3.11 $$\begin{aligned} \bigl\vert u'(t)\bigr\vert \leq&\lambda\bigl|F \bigl(u(t)\bigr)-F\bigl(u(t_{2})\bigr)\bigr|+\lambda \int _{t_{2}}^{t_{2}+T}g\bigl(u(t)\bigr)\,dt \\ &{}+\lambda \int_{t_{2}}^{t_{2}+T}\bigl|\varphi (s)\bigr|u(s)\,ds+\lambda \int_{t_{2}}^{t_{2}+T}\bigl|h(s)\bigr|\,ds \\ \leq& 2\lambda\max_{0\leq u\leq M_{1}}\bigl|F(u)\bigr|+\lambda \int _{0}^{T}g\bigl(u(s)\bigr)\,ds+\lambda T \overline{|\varphi|}|u|_{\infty}+\lambda T\overline{|h|}. \end{aligned}$$

From (3.2), we see that

$$\begin{aligned} \int_{0}^{T}g\bigl(u(s)\bigr)\,ds =& \int_{0}^{T}\varphi(t)u(t)\,dt-T\bar {h} \\ \le&T\overline{\varphi_{+}}|u|_{\infty}+T\overline{h_{-}}. \end{aligned}$$

It follows from (3.10) and (3.11) that

3.12 $$\begin{aligned} &\bigl\vert u'(t)\bigr\vert \leq2\lambda \Bigl( \max_{0\leq u\leq M_{1}}\bigl\vert F(u)\bigr\vert + T\overline{| \varphi|}|u|_{\infty}+T\overline{|h|} \Bigr) \\ &\quad< 2\lambda \Bigl( \max_{0\leq u\leq M_{1}}\bigl|F(u)\bigr|+ M_{1}T \overline{|\varphi |}+T\overline{|h|} \Bigr) \\ &\quad:=\lambda M_{2},\quad t\in[0,T], \end{aligned}$$

and then

3.13 $$ \bigl\vert u'(t)\bigr\vert < M_{2}, \quad\mbox{for all } t\in[0,T]. $$

Equations (3.10) and (3.13) imply that (3.1) holds.

Below, we will show that there exists a constant $\gamma_{0}\in (0,\gamma)$, such that each positive *T*-periodic solution of equation (2.1) satisfies

3.14 $$ u(t)>\gamma_{0} \quad\mbox{for all } t\in[0,T]. $$

Suppose that $u(t)$ is an arbitrary positive *T*-periodic solution of equation (2.1), then

3.15 $$ u''+\lambda f(u)u'-\lambda g(u)+\lambda\varphi(t)u=\lambda h(t),\quad \lambda\in(0,1]. $$

Let $t_{1}$ be determined in (3.6). Multiplying (3.15) by $u'(t)$ and integrating it over the interval $[t_{1},t]$ (or $[t,t_{1}]$), we get

$$\frac{|u'(t)|^{2}}{2}-\frac{|u'(t_{1})|^{2}}{2}+\lambda \int _{t_{1}}^{t}f(u) \bigl(u' \bigr)^{2}\,dt=\lambda \int_{t_{1}}^{t}g(u)u'\,dt-\lambda \int _{t_{1}}^{t}\varphi(t)uu'\,dt+\lambda \int_{t_{1}}^{t} h(t)u'\,dt, $$

which yields the estimate

$$\begin{aligned} \lambda\biggl\vert \int_{u(t)}^{u(t_{1})}g(s)\,ds\biggr\vert \leq{}& \frac {|u'(t)|^{2}}{2}+\frac{|u'(t_{1})|^{2}}{2}+\lambda \int _{0}^{T}\bigl\vert f(u)\bigr\vert \bigl(u'\bigr)^{2}\,dt \\ &{}+\lambda \int_{0}^{T}\bigl\vert \varphi (t)uu' \bigr\vert \,dt+\lambda \int_{0}^{T} \bigl\vert h(t)u'\bigr\vert \,dt. \end{aligned}$$

From (3.10) and (3.12), we get

$$\lambda\biggl\vert \int_{u(t)}^{u(t_{1})}g(s)\,ds\biggr\vert \leq\lambda M_{2}^{2}+\lambda\max_{0\leq u\leq M_{1}}\bigl\vert f(u)\bigr\vert TM_{2}^{2}+\lambda M_{1}M_{2}T \overline{|\varphi|}+\lambda M_{2}T\overline{|h|}, $$

which gives

3.16 $$ \biggl\vert \int_{u(t)}^{u(t_{1})}g(s)\,ds\biggr\vert \leq M_{3}, \quad\mbox{for all } t\in[t_{1},t_{1}+T], $$

with

$$M_{3}= M_{2}^{2}+\max_{0\leq u\leq M_{1}} \bigl\vert f(u)\bigr\vert TM_{2}^{2}+ M_{1}M_{2}T \overline {|\varphi|}+ M_{2}T\overline{|h|}. $$

From [H_3_] there exists $\gamma_{0}\in(0,\gamma)$ such that

3.17 $$ \int_{\eta}^{\gamma}g(u)\,du>M_{3}, \quad\mbox{for all } \eta\in(0,\gamma_{0}]. $$

Therefore, if there is a $t^{*}\in[t_{1},t_{1}+T]$ such that $u(t^{*})\le\gamma _{0}$, then from (3.17) we get

$$\int_{u(t^{*})}^{\gamma}g(s)\,ds> M_{3}, $$

which contradicts (3.16). This contradiction gives that $u(t)>\gamma_{0}$ for all $t\in[0,T]$. So (3.14) holds. Let $m_{0}=\min\{D_{1},\gamma_{0}\}$ and $m_{1}\in(M_{1}+D_{2}, +\infty)$ be two constants, then from (3.1) and (3.14), we see that each possible positive *T*-periodic solution *u* to equation (2.1) satisfies

$$m_{0}< u(t)< m_{1},\quad \bigl\vert u'(t) \bigr\vert < M_{2}. $$

This implies that condition 1 and condition 2 of Lemma 1 are satisfied. Also, we can deduce from Remark 1 that

$$g(c)-\bar{\varphi}c+\bar{h}>0, \quad\mbox{for } c\in(0, m_{0}] $$

and

$$g(c)-\bar{\varphi}c+\bar{h}< 0, \quad\mbox{for } c\in[ m_{1},+\infty), $$

which results in

$$\bigl(g(m_{0})-\bar{\varphi}m_{0}+\bar{h} \bigr) \bigl(g(m_{1})-\bar {\varphi}m_{1}+\bar{h} \bigr)< 0. $$

So condition 3 of Lemma 1 holds. By using Lemma 1, we see that equation (1.1) has at least one positive *T*-periodic solution. The proof is complete. □

Let us consider the equation

3.18 $$ x''+f(x)x'- \frac{1}{x^{\gamma}}+\varphi(t)x=h(t), $$

where $f : [0,+\infty)\rightarrow R$ is an arbitrary continuous function, $\varphi,h: R\rightarrow R$ are *T*-periodic functions with $h\in L^{1}([0,T], R)$ and $\varphi\in C([0,T], R)$, and the sign of the function *φ* is allowed to change for $t\in[0,T]$, $\gamma \ge1$ is a constant. Corresponding to equation (1.1), $g(x)=\frac{1}{x^{\gamma}}$. For this case, $g(x)\rightarrow+\infty$ as $x\rightarrow0^{+}$, and assumptions [H_2_]-[H_3_] are satisfied. Thus, by using Theorem 1, we have the following results.

### Corollary 1

*Assume that the function* $\varphi(t)$ *satisfies the following conditions*:

$$\int_{0}^{T}\varphi_{+}(s)\,ds>0, \qquad \sigma:= \frac{\int_{0}^{T}\varphi_{-}(s)\,ds}{\int _{0}^{T}\varphi_{+}(s)\,ds}\in[0,1) $$

*and*

$$\sigma_{1}:=\frac{T}{\pi}|\varphi_{+}|_{\infty}^{1/2}+ \frac{T^{\frac {1}{2}} (\int_{0}^{T}\varphi_{-}(s)^{2}\,ds )^{\frac{1}{2}}}{\int _{0}^{T}\varphi_{+}(s)\,ds}\in(0,1). $$

*Then*, *equation* (3.18) *possesses at least one positive* *T*-*periodic solution*.

### Remark 2

Corresponding to equation (1.4) and equation (1.5), the function $g(t,x)$ associated to equation (3.18) can be regarded as

3.19 $$ g(t,u)=-\frac{1}{u^{\gamma}}+\varphi(t)u-h(t),\quad (t,u)\in[0,T] \times(0,+\infty). $$

For the case of $\varphi(t)\ge0$ for all $t\in[0,T]$, we see that if *x* is a positive *T*-periodic continuous function satisfying $\int _{0}^{T} g(t,x(t))\,dt=0$, then

3.20 $$ \int_{0}^{T}\frac{1}{x^{\gamma}(t)}\,dt= \int _{0}^{T}\varphi(t)x(t)\,dt- \int_{0}^{T}h(t)\,dt. $$

By applying the integral mean value theorem to the term $\int _{0}^{T}\varphi(t)x(t)\,dt$ in equation (3.20), one can easily verify that $g(t,u)$ determined in (3.19) satisfies the balance condition (h_1_). However, if the sign of the function $\varphi(t)$ is changeable for $t\in[0,T]$, then it is unclear from (3.20) whether the balance condition (h_1_) is satisfied. For this case, the main results of [18, 19] cannot be applied to equation (3.18).

### Corollary 2

*Assume that the function* $\varphi(t)$ *satisfies* $\varphi(t)\ge0$ *for all* $t\in[0,T]$ *with* $\int_{0}^{T}\varphi(s)\,ds>0$, *and*

$$|\varphi|_{\infty}< \biggl(\frac{\pi}{T} \biggr)^{2}. $$

*Then*, *equation* (3.18) *possesses at least one positive* *T*-*periodic solution*.

### Example 1

Consider the following equation:

3.21 $$ x''(t)+f\bigl(x(t)\bigr)x'(t)- \frac {1}{x^{2}(t)}+a(1+2\sin2t)x(t)=\cos2t, $$

where *f* is an arbitrary continuous function, $a\in(0,+\infty)$ is a constant. Corresponding to equation (3.18), we have $\gamma=2$, $\varphi(t)=a(1+2\sin2t)$ and $h(t)=\cos2t$, $T=\pi$. By simply calculating, we can verify that

$$\begin{aligned}& \int_{0}^{T}\varphi_{+}(t)\,dt=\biggl( \frac{2\pi}{3}+\frac {3}{2}\biggr)a,\qquad \int_{0}^{T}\varphi_{-}(t)\,dt=\biggl( \frac{3}{2}-\frac{\pi}{3}\biggr)a, \\& \int_{0}^{T}\bigl(\varphi_{-}(t)\bigr)^{2}\,dt= \frac{3\pi a}{2}, \end{aligned}$$

and then

$$\sigma:=\frac{\int_{0}^{T}\varphi_{-}(s)\,ds}{\int_{0}^{T}\varphi_{+}(s)\,ds}=\frac {9-2\pi}{4\pi+9}\in(0,1) $$

and

$$\sigma_{1}:=\frac{T}{\pi}|\varphi_{+}|_{\infty}^{1/2}+ \frac{T^{\frac {1}{2}} (\int_{0}^{T}\varphi_{-}(s)^{2}\,ds )^{\frac{1}{2}}}{\int _{0}^{T}\varphi_{+}(s)\,ds}=\sqrt{3a}+\frac{3\pi\sqrt{6}}{4\pi+9}. $$

Thus, if $0< a<\frac{1}{3} (\frac{4\pi+9-3\pi\sqrt{6}}{4\pi+9} )^{2}$, then $\sigma_{1}\in(0,1)$. By using Corollary 1, we see that equation (3.21) has at least one positive *π*-periodic solution.

### Remark 3

Since the sign of $\varphi(t)=1+2\sin t$ is changed for $t\in[0,T]$, whether the right inequality of (1.6) in the balance condition (h_1_) is satisfied remains unclear. So the conclusion of the example cannot be obtained by using the main results in [18, 19].
